# Longitudinal Imaging of the Foveal Cone Mosaic in *CNGA3*-Associated Achromatopsia

**DOI:** 10.1167/iovs.65.12.6

**Published:** 2024-10-04

**Authors:** Mohamed Katta, Michalis Georgiou, Navjit Singh, Angelos Kalitzeos, Alfredo Dubra, Joseph Carroll, Michel Michaelides

**Affiliations:** 1 UCL Institute of Ophthalmology, University College London, London, United Kingdom; 2 Moorfields Eye Hospital NHS Foundation Trust, London, United Kingdom; 3 Department of Ophthalmology, Stanford University, Palo Alto, California, United States; 4 Department of Ophthalmology and Visual Sciences, Medical College of Wisconsin, Milwaukee, Wisconsin, United States

**Keywords:** adaptive optics, inherited retinal disease (IRD), CNGA3, achromatopsia (ACHM), cone dysfunction

## Abstract

**Purpose:**

The purpose of this study was to assess the natural history of the foveal cone mosaic in *CNGA3*-associated achromatopsia (ACHM).

**Methods:**

Thirteen eyes from 10 genetically confirmed patients underwent longitudinal imaging with optical coherence tomography (OCT) and non-confocal split detection adaptive optics scanning light ophthalmoscopy (AOSLO). OCT scans assessed outer nuclear layer (ONL) thickness, foveal ellipsoid zone (EZ) disruption, and foveal hypoplasia. AOSLO images were analyzed to calculate peak foveal cone density (PCD) and mean inter-cell distance (ICD) between cones. Mixed effects models were used to analyze the rate of annual change of PCD and ICD.

**Results:**

Mean (±SD) age at visits was 29 ± 10 years, with a follow-up of 2.6 ± 1 years. There was no change in ONL thickness, degree of EZ disruption, or foveal hypoplasia over the follow-up period. We also observed a stable foveal cone mosaic using AOSLO imaging, with no significant change in PCD or ICD. Mean PCD was 15,346 cones/mm² at the mean age of 29 years old (cf. 64,000–324,000 cones/mm² in previously reported healthy controls), with a mean rate of change of –117.79 cones/mm² (0.8%) per year, *P* = 0.130. Mean ICD at the mean age was 13.82 µm, with a rate of change of 0.17 µm per year, *P* = 0.83.

**Conclusions:**

*CNGA3*-associated ACHM displays stable foveal cone structure over time with a similar rate of change to *CNGB3*-associated ACHM (2% decline per year). The stable PCD, small cohort, and large variability within the cohort means significant age associations were not detected.

Achromatopsia (ACHM) is an autosomal recessive inherited retinal disorder (IRD) affecting cone function. The majority of causative variants are found in the *CNGB3* and *CNGA3* genes, both of which are being targeted in several gene therapy trials (*CNGB3*: NCT03001310 and NCT02599922 and *CNGA3*: NCT03758404, NCT02610582, and NCT02935517).^1^^,^^2^ Reduced visual acuity, absent color vision, nystagmus, and photophobia are key features of the condition.^3^ ACHM is viewed as a largely non-progressive stationary disease with stable visual acuity and color vision.^4^ One aspect of the disease which is attractive as a gene therapy candidate is the relatively intact foveal retinal structure compared to other IRDs, which is stable over time, thus giving a large potential treatment window. The majority of retinal imaging studies in ACHM have been conducted on patients with *CNGB3* as this is the most prevalent genotype.^3^^,^^5^^,^^6^

The synopsis of longitudinal OCT imaging studies is that ACHM shows structural changes which remain stationary over time in the outer retina,^7^^–^^9^ or, at worst, very slow progression.^10^ The ellipsoid zone (EZ) that forms the inner/outer photoreceptor segment junction can range from seemingly normal morphology to varying degrees of disruption in ACHM.^7^^,^^11^^–^^14^ In a cross-sectional study of 40 patients with ACHM, Sundaram et al.^7^ reported no age-related association with outer nuclear layer (ONL) thickness or outer retinal disruption. Similarly, Langlo et al.^15^ did not find an association between age and ONL thickness but reported a significant increase in mean age between worsening EZ OCT grades of ACHM. Thomas et al.^11^ and Thiadens et al.^12^ also reported worse outer retinal structure with age in ACHM in cross-sectional studies.

Building on these studies, imaging with non-confocal adaptive optics scanning light ophthalmoscopy (AOSLO) has allowed the characterization of the foveal cone mosaic in ACHM by identifying individual cone photoreceptors in both *CNGB3* and *CNGA3* genotypes,^6^^,^^15^^,^^16^ as well as the rarer genotypes of *ATF6*, *GNAT2*, and *PDE6C*.^17^^–^^19^ Visualizing the cone photoreceptors in vivo may prove to be a useful tool for defining the therapeutic benefit of candidate treatments and potentially stratifying patients by treatment prognosis using foveal mosaic metrics to better inform patient selection for clinical trials. AOSLO imaging may provide an additional non-redundant means of assessing outer retinal integrity alongside OCT imaging as the two imaging modalities have been shown to be uncorrelated.^16^^,^^20^ In a small study comparing OCT with AOSLO imaging in five patients, Scoles et al. found that EZ integrity was not a good predictor for the presence of photoreceptors and further Georgiou et al. reported no correlation between ONL thickness from OCT and peak cone density (PCD) from AOSLO imaging of patients with *CNGA3*.^16^^,^^20^ The inter-ocular symmetry of PCD in patients with ACHM also provides a suitable control eye for the treated eye in clinical trials.^21^ The utility of AOSLO imaging is predicated on understanding the natural history of the foveal mosaic in ACHM and delineating any genotype-phenotype relationships that may exist.

Literature on *CNGA3*-ACHM imaging with AOSLO is limited, with no longitudinal studies reported to date. Langlo et al.^22^ reports the only other longitudinal study on patients with *CNGB3*, reporting an overall stable foveal cone mosaic with a 2% decline in PCD per year in their cohort. The purpose of this study is to perform the first longitudinal AOSLO study of *CNGA3*-ACHM, with comparison to longitudinal OCT metrics, building on a previous cross-sectional study.^16^ This will provide insight on the natural history of the *CNGA3*-ACHM foveal cone mosaic and enable comparison to reported cone mosaic changes in patients with *CNGB3*-ACHM.

## Methods

This study was approved by the ethics committee of Moorfields Eye Hospital and the health research authority ethics committee (11/LO/1229). Written informed consent was obtained from all patients after explanation of the nature and possible consequences of the study. The research followed the tenets of the Declaration of Helsinki.

Fifteen patients with ACHM with disease-causing variants in *CNGA3* who participated in a prior cross-sectional study were recruited into the longitudinal study from Moorfields Eye Hospital, London, UK.^16^ Of those initially recruited, images from 10 patients imaged at 2 to 3 time points (4 patients with 2 time points and 6 patients with 3 time points), spaced a year apart, were of high enough quality to be included in the longitudinal analysis.

Exclusion criteria were patients with either nystagmus or low signal to noise ratio images. Only those patients with at least two imaging sessions covering the entire rod-free zone at least a year apart were included. Two patients were lost to follow-up and three patients did not have repeat images of high enough quality for analysis due to nystagmus. Pupil dilation prior to imaging was achieved with Tropicamide 1% and Phenylephrine 2.5% eye drops.

### Spectral-Domain-OCT Imaging

Spectral-domain OCT (SD-OCT) imaging was performed on the Spectralis system (Heidelberg Engineering Inc., Heidelberg, Germany). Volumetric horizontal SD-OCT scans were analyzed for structural retinal changes using the HEYEX2 software by a single grader (author M.K.) twice, for both the first and last visits in the follow-up series, then the quantitative data were averaged. Scans were automatically segmented for the different retinal layers and then corrected manually if required. Foveal ONL thickness was measured by setting the display ratio to 1:1 µm on the transfoveal line scan passing through the center of the foveal pit. The measurement was taken at the fovea from the border of the inner limiting membrane (ILM) and the external limiting membrane (ELM). For scans showing foveal hypoplasia (persistence of the inner retinal layers over the fovea), the measurement was taken from the outer plexiform-ONL border to the ELM. Patients with foveal hypoplasia were noted. Transfoveal line scans were also used to grade the integrity of the EZ according to the published grading system described by Sundaram et al.^7^ The grades, are as follows: (1) a continuous EZ, (2) EZ disruption, (3) EZ absence, (4) presence of a hyporeflective zone (HRZ), and (5) outer retinal atrophy with retinal pigment epithelium loss. A similar OCT imaging protocol has been used by several ACHM imaging studies,^7^^,^^15^^,^^23^ and is the same protocol used in the cross-sectional ACHM AOSLO imaging study that this longitudinal study builds on.^16^

### AOSLO Imaging of Cone Photoreceptor Mosaic

Confocal reflectance and non-confocal split-detection image sequences were acquired simultaneously in absolute temporal and spatial registration. Non-confocal split-detection allows imaging of the photoreceptor inner segment mosaic, which in turn allows cones with compromised outer segments to be visualized.^24^ The AOSLO light source was a 790 nm super-luminescent diode (SLD; Superlum, Carrigtwohill, Cork, Ireland). Image sequences consisting of 150 frames at either 1- or 1.5-degrees field of view were recorded at each retinal location. Sinusoidal distortions caused by the horizontal scanner were compensated for using a Ronchi ruling, as previously described.^25^ A representative frame for each retinal location with the least amount of distortion was then selected as reference against which at least 40 frames were registered and averaged to increase the signal to noise ratio.^26^ Images were then stitched together into a final montage of the imaged retina using custom, open-source software.^27^ Image scales were computed using each patient's axial length (IOLMaster 500, Carl Zeiss Meditec AG, Jena, Germany) to correct for ocular magnification accordingly and the final micron per pixel scale calculated by multiplying by the retinal magnification factor of 291 µm/degree. The anatomic foveal center was identified by the characteristic non-waveguiding rod-free zone in corresponding confocal AOSLO images, as described by Langlo et al.^15^ in Supplementary Figure S1, and montages were aligned across time points for analysis.

The center of every cone was annotated manually by a single grader (author M.K.) twice. A 55 µm square sampling window was used to derive the PCD (cones/mm²) and its location using PeakDensityAnalytics (available at DOI: 10.5281/zenodo.10823302), a custom, open-source software in MATLAB (Math-Works, Natick, MA, USA).^27^ Mean intercell-distance (ICD), the mean distance of each cone to its adjacent cones was analyzed for the rod free zone and used as a measure of the packing of residual cone inner segments.^28^ PCD and ICD values for the two counts performed were then averaged.

### Statistical Methods

IBM SPSS statistics (version 29.0; IBM Corp., Armonk, NY, USA) was used for all statistical analyses. The intraclass correlation coefficient (ICC) of ONL measurement and PCD averaged data over the duplicate measurements by a single grader was calculated to assess intrarater agreement between the two measurements. Paired *t*-tests were used for assessing potential ONL thickness change between the first and last visits after testing for normality (Shapiro-Wilk test), and homogeneity of variance. Mixed effects models were used to analyze PCD and ICD data. Analyses were run with PCD or ICD as the dependent variable with patients as the random factor. Random intercept and slopes for each patient were incorporated into the models. A conditional ICC was produced for each mixed effect model analysis. Conditional ICCs closer to 1 indicate greater intrapatient rather than interpatient variability over time in the dependent variable (PCD or ICD) explains the total variance in the model. Statistical significance was considered at *P* values less than 0.05.

## Results

### Demographics

Thirteen eyes of 10 patients (7 female patients) from 9 different families were included in the final analysis; with a mean (± SD) age at the first visit of 27 ± 10.3 years (range = 14 – 55 years). The mean (± SD) follow-up period was 2.6 ± 1 years. There was one pair of siblings and two pairs of unrelated patients that shared a genetic variant. The demographics and disease-causing variants are summarized in Supplementary Table S1.

### SD-OCT Imaging

The retinal morphology findings at each visit are shown in the Table. One patient (10%) had a continuous EZ (grade 1), five patients (50%) had grade 2, and four patients (40%) had grade 4. There were no patients with an absent EZ (grade 3) or retinal atrophy (grade 5). There was no change in EZ grading for any of the patients over time. There was no significant change in ONL thickness over the period of follow-up, with mean (± SD) thickness at the first visit of 54.15 ± 17.58 µm and 54.23 ± 17.79 µm at the final visit (t = –0.97, *P* = 0.925, paired *t*-test).The mean (± SD) ONL thickness across visits by grade were for grade 1 = 55 ± 3.0 µm, grade 2 = 46.9 ± 18.5 µm, and grade 4 = 60.3 ± 16.7 µm. Foveal hypoplasia was seen in six patients (60%) and there was no change in the morphology over time. The repeatability of ONL thickness measurements was high between the two measurements taken for each time point with an ICC of 0.98 (95% confidence interval 0.95–0.99).

**Table.** Summary of SD-OCT and AOSLO Retinal Morphology

Subject	OS/OD	Age at Visit, Y	Foveal Hypoplasia	EZ Grade	ONL Thickness, µm	Mean PCD, Cones/mm²	Mean ICD, µm
MM_0014	OD	34.72	Yes	4	77	4,663	23.72
MM_0014	OS	34.72	Yes	4	76	5,216	24.32
MM_0014	OD	35.73	Yes	4	76	4,354	24.05
MM_0014	OS	35.73	Yes	4	74	4,784	24.78
MM_0014	OD	36.72	Yes	4	72	4,678	23.55
MM_0014	OS	36.72	Yes	4	79	5,077	25.13
MM_0015	OD	28.08	Yes	4	43	22,708	13.74
MM_0015	OS	28.08	Yes	4	49	28,768	11.56
MM_0015	OD	29.31	Yes	4	41	22,904	13.81
MM_0015	OS	29.31	Yes	4	45	28,875	11.42
MM_0064	OD	23.84	No	2	26	15,067	11.76
MM_0064	OS	23.84	No	2	26	12,288	12.55
MM_0064	OD	25.94	No	2	25	13,884	11.54
MM_0064	OS	25.94	No	2	28	12,726	12.04
MM_0064	OD	27.09	No	2	25	16,337	11.09
MM_0064	OS	27.09	No	2	26	12,149	12.59
MM_0170	OS	16.70	Yes	2	56	32,661	7.57
MM_0170	OS	17.70	Yes	2	57	32,754	7.66
MM_0170	OS	19.84	Yes	2	60	32,640	7.75
MM_0385	OD	37.70	No	4	38	5,389	22.20
MM_0385	OD	38.88	No	4	41	5,476	21.47
MM_0385	OD	39.70	No	4	39	5,312	23.11
MM_0386	OD	22.21	Yes	2	64	11,426	13.06
MM_0386	OD	23.23	Yes	2	63	11,172	13.17
MM_0398	OD	14.40	Yes	2	51	9,033	13.61
MM_0398	OD	16.39	Yes	2	54	9,175	14.71
MM_0418	OD	18.32	No	2	72	32,624	6.89
MM_0418	OD	21.64	No	2	70	31,838	6.88
MM_0446	OS	50.99	Yes	4	71	6,742	17.86
MM_0446	OS	52.07	Yes	4	71	6,956	17.17
MM_0446	OS	54.75	Yes	4	73	6,900	17.32
MM_0458	OS	19.27	No	1	55	22,441	8.39
MM_0458	OS	20.25	No	1	52	22,594	8.12
MM_0458	OS	23.43	No	1	58	20,601	8.85

### AOSLO Imaging of Foveal Mosaic

**Figure 1.**
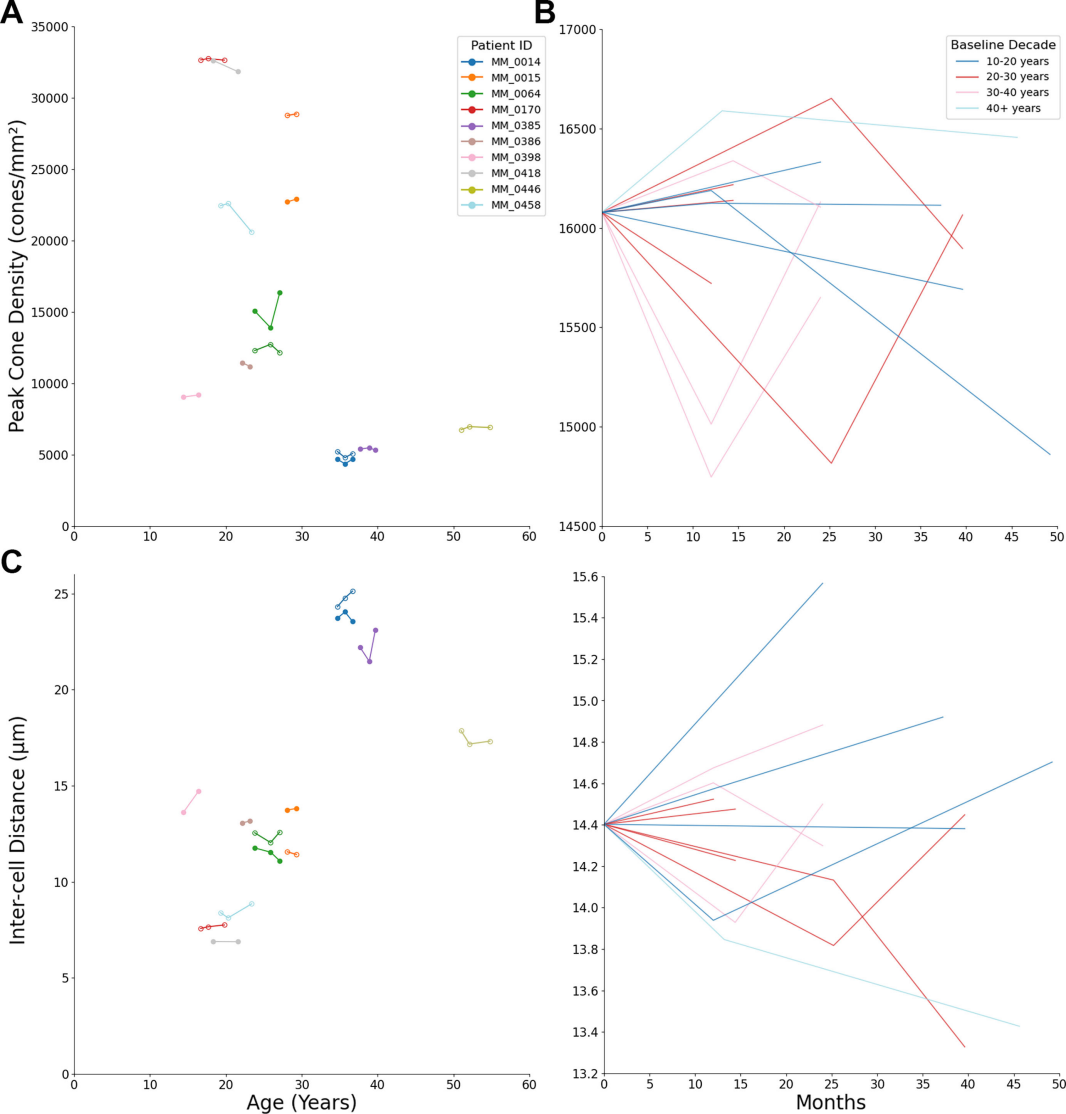
**Peak cone density (PCD) and inter-cell distance (ICD) of each eye over the follow-up period.** (**A,** **C**) PCD and ICD by age, (**B,** **D**) PCD and ICD by months of follow-up normalized to the mean baseline of 16,079 cones/mm² for PCD and 14.4 µm for ICD, and color coded by age at baseline. The younger patients showed greater decline in PCD. OD = *solid circles*, OS = *open circles*.

There was no statistically significant difference between the PCD or ICD of the baseline or last visit for each patient. Baseline mean (± SD) PCD was 16,079 ± 10,530 cones/mm^2^, with a final visit PCD mean (± SD) of 15,974 ± 10,375 cones/mm^2^; paired *t*-test; *t*(13) = 0.55, *P* = 0.3. Baseline mean (± SD) ICD was 14.4 ± 5.9 µm, with a final visit ICD mean of 14.6 ± 6.15 µm, paired *t*-test; *t*(13) = –1.13, *P* = 0.14. The estimated marginal mean PCD was calculated to be 15,346 cones/mm² at the mean age of 29 years old (SE = 2605), with a rate of change of –118 cones/mm² per year (SE = 76, conditional ICC = 0.98, df = 32, F = 2.4, *P* = 0.13). The estimated marginal mean ICD was 13.8 µm (SE = 21.6), with a rate of change of 0.17 µm (SE = 0.75, conditional ICC = 0.99, df = 32, F = 0.49, *P* = 0.83). Figure 1 shows the PCD and ICD of each eye over the follow-up period, younger patients tended to have a greater change in PCD compared with older patients. Patient MM_0458 showed the largest absolute decrease in PCD of 9.2% over 4 years (22,441 to 20,601 cones/mm²).

**Figure 2.**
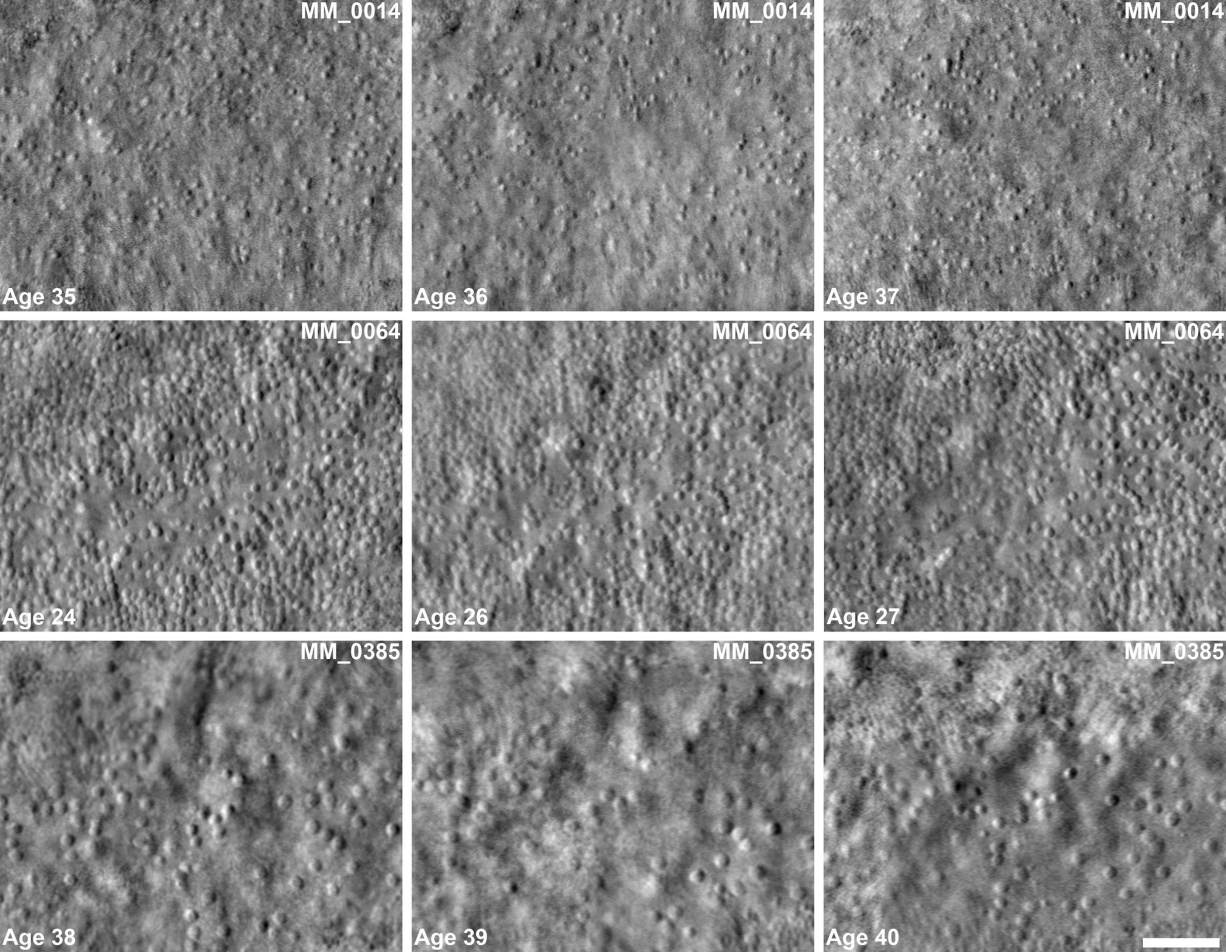
**Longitudinal AOSLO imaging of the foveal cone mosaic.** Examples of three patients with longitudinal AOSLO imaging of the foveal cone mosaic. No large-scale changes in the foveal cone mosaic are noted over follow-up. Scale bar = 50 µm. PCDs left to right for MM_0014 = 4663, 4354, and 4678 cones/mm², MM_0064 = 15,067, 13,884, and 16,337 cones/mm², MM_0385 = 5389, 5476, and 5312 cones/mm².

**Figure 3.**
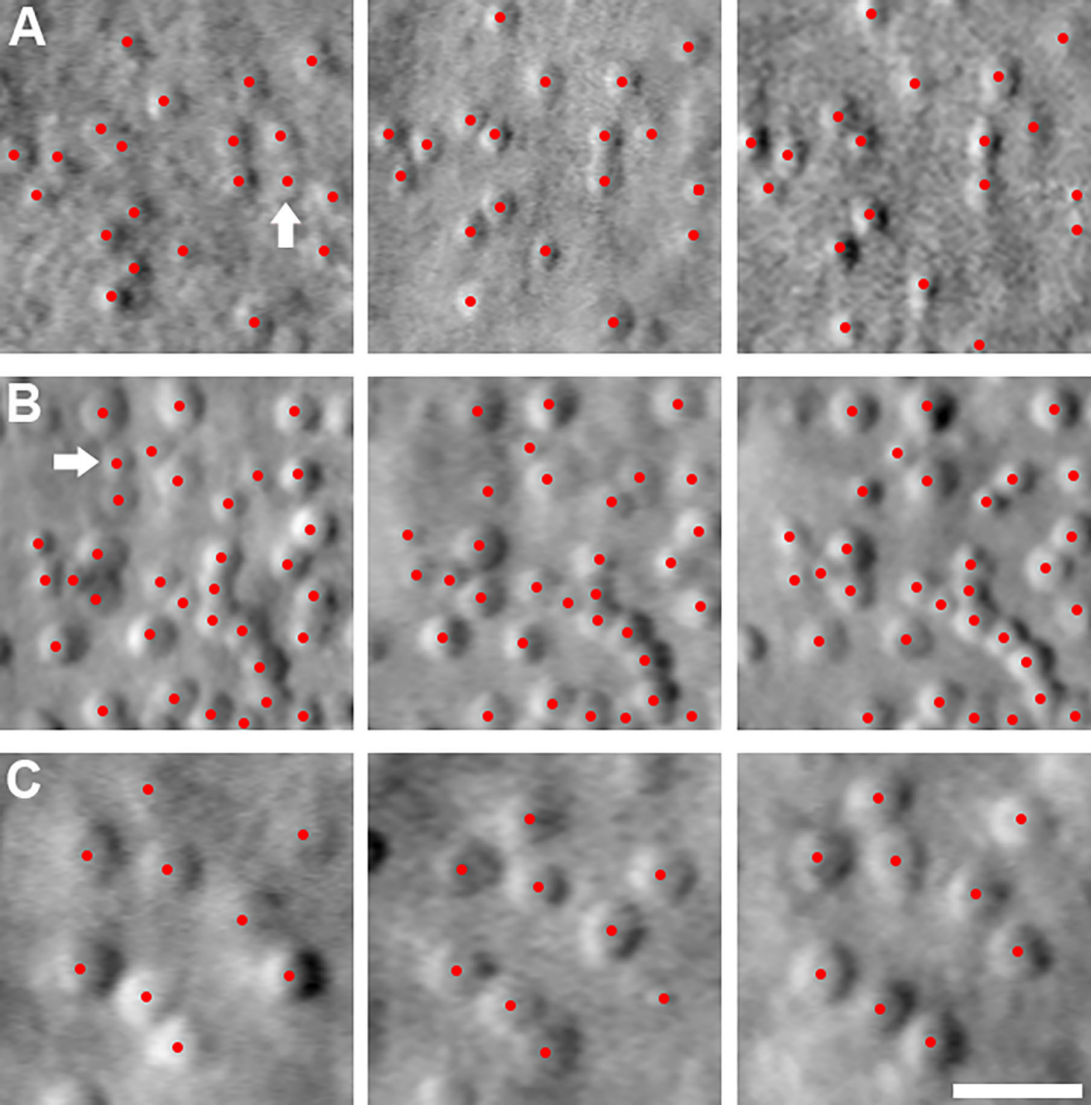
**Regions of interest in three patients over** **the** **follow-up period.** (**A**) MM_0014 aged 35, 36, and 37 years, (**B**) MM_0064 aged 24, 26, and 27 years, and (**C**) MM_0385 aged 38, 39, and 40 years, respectively. The *arrowed cones* in rows A and B are not counted in the subsequent two time points which most likely represent true cone loss. Scale bar = 20 µm.

Examination of the serial AOSLO imaging depicted in Figure 2 qualitatively shows no obvious change over time. Figure 3 shows examples of stable cone counts in 3 patients over the follow-up period in a region of interest. The repeatability of PCD measurements was high between the 2 measurements taken at each imaging visit with an ICC of 0.96 (95% confidence interval 0.90–0.98).

### Intra-Familial and Intra-Variant Differences

Due to the small number of patients within familial and variant subgroups, statistical analysis was not applied. There was a large difference between the mean PCD across follow-up of the siblings in the cohort despite their similar age at imaging and the same OCT EZ grading of grade 4: MM_0014 (4795 cones/mm² at 36 years) versus MM_0015 (25,813 cones/mm² at 29 years). Similarly, there was a large variation between unrelated patients who shared a common variant despite the same EZ grading. Two unrelated patients carried the c*.661C>T*, p.Arg221Ter variant and both had an EZ grading of grade 2, MM_0064 (13,742 cones/mm² at 26 years) and MM_0418 (32,231 cones/mm² at 20 years). Two other unrelated patients carried the c.67C>T, p.Arg23Ter variant, MM_0170 (32,685 cones/mm² at 18 years) and MM_0446 (6866 cones/mm² at 53 years), but with different EZ grading of grades 2 and 4, respectively. None of these patients had significant changes in PCD over the follow-up period, as shown in the Table.

## Discussion

*CNGA3*-associated ACHM displays a relatively stable cone mosaic structure over time with a nonsignificant trend toward a slow change of PCD of 0.62% annually. AOSLO studies using healthy subjects have found variable relationships between cone density and age. In a study assessing 29 healthy controls with AOSLO, Baraas et al. found no association between cone density and age (range = 15–70 years).^29^ In a smaller cohort, Chui et al. compared 2 groups of 8 healthy controls with mean ages of 27 and 56 years imaged with AOSLO, and found a small (4%) but significant decrease in mean cone density between the younger and older groups.^30^ In a longitudinal study examining parafoveal eccentricities, cone density was found to be stable in a healthy adult cohort over a 2-year period of imaging.^31^ In a cross-sectional study of *CNGB3*-ACHM, Langlo et al.^15^ found no association between age and PCD. In a further longitudinal study of *CNGB3*-ACHM, Langlo et al.^22^ found a PCD rate of 2% per year in a cohort of 18 patients. This is more than twice the decay rate found in this *CNGA3* cohort (0.8% per year). In contrast, Georgiou et al. in the cross-sectional study that included patients from this study found a negative correlation between age and PCD.^16^ Indeed, younger patients in this study were also observed to have a greater PCD at baseline compared with older patients but taken at a single time point, these results would suggest significant foveal cone change.

However, the findings in this study herein are from the same aforementioned patients over time and therefore a greater degree of confidence can be taken when extrapolating observed results over age compared to results from cross-sectional studies. Younger patients were estimated to have a greater decay in cones than older groups which may be related to their higher baseline PCD. The patient with the greatest PCD decay rate of 2.3% per year, MM_0458, was also one of the youngest at 19 years old at baseline. Similarly, Langlo et al.^15^ found that patients with *CNGB3* with a baseline PCD below 25,000 cones/mm² had relatively stable densities, whereas those above this density showed more cone decay. This may indicate that in both *CNGA3*- and *CNGB3*-associated ACHM, cone decay is not linear with a more rapid component in early life that then stabilizes with age. The PCD decay rates in both the *CNGB3* cohort and this *CNGA3* cohort were not statistically significant and observed differences in decay rates between the two genotypes may be due to the small cohort sizes and large interindividual variability in PCD between patients with ACHM. These findings fit well into those previously reported of a robust, cone mosaic in ACHM with possible slow age-related decline in PCD similar to healthy foveas. In a histological study of 27 healthy foveas mounted shortly after death (range = 27–90 years old), Curcio et al. found an approximate mean PCD of 175,000 cones/mm² using the regression equation provided and 197,000 cones/mm² at age 29 years (the mean age of this study), with a nonsignificant loss of 716 cones/mm² (0.4%) per year, see for example, 15,346 cones/mm², –118 cones/mm² (0.8%) per year in this study.^32^ Therefore any observed decay in cones in adult patients with ACHM may simply be due to aging rather than disease progression.

Similar to other studies in patients with *CNGB3* and *CNGA3*, there was great interindividual variability in outer retinal structure graded by OCT.^15^^,^^16^^,^^33^ Mean ONL thickness was also thinner in this cohort compared with these larger previously reported *CNGB3* and *CNGA3* cohorts, despite a similar rate of foveal hypoplasia (60% this study versus 71% Langlo et al. and Georgiou et al.), and similar mean age of cohorts (29 ± 10 years this study versus 23 ± 10 years in Langlo et al., 24 ± 14 years in Mastey et al., and 28 ± 14 years in Georgiou et al.).^15^^,^^16^^,^^33^ This difference is likely due to the smaller number of patients in this longitudinal study. ONL thickness in ACHM has been reported to decrease,^23^^,^^34^ stay stable,^7^^,^^10^ and slightly increase over time by different groups.^22^ In agreement with the previous *CNGA3* cross-sectional study,^16^ ONL thickness remained stable in this cohort over the follow-up period.

There was significant variability in PCD within siblings and unrelated patients carrying the same genetic variants. Thus, it appears there is no discernible foveal cone phenotype with respect to genotype. This wide variability in PCD between ages and variants makes it difficult to determine a therapeutically useful cutoff age for potential gene therapy candidates based on cone integrity alone.

There are some inherent limitations to this study. First, the rarity of ACHM and the fact that the *CNGA3* genotype is less common than *CNGB3*,^35^ makes assessment of large cohorts difficult. Second, the follow-up period of the study is relatively short with a mean follow-up time of 2.5 years, the lack of significant progression may be partly due to this short assessment period and slow natural history of the disease. Of the 15 patients who took part in the original cross-sectional study, only 10 (67%) could consistently be imaged to the high quality required to allow PCD assessment. This imaging success rate was higher than that reported by others in patients with ACHM of 44% to 54%.^15^^,^^16^^,^^22^^,^^36^ This is due to the prior selection of these patients following the cross-sectional study.^16^ Failure to achieve analyzable images was mostly due to nystagmus, which can be variable within the same patient on different sessions and/or between eyes. Nystagmus in ACHM has been reported to improve over time.^37^^,^^38^ This improvement in nystagmus, combined with the overall higher level of patient compliance required for successful AOSLO imaging sessions that sometimes take upward of 2 hours to perform, means analyzable images are more likely to be obtained with adult patients. Indeed, the youngest patient in this cohort is 14 years old at baseline. This leads to reported AOSLO studies of ACHM including this study having an older patient cohort, and we, therefore, have to be cautious when extrapolating to younger age ranges that are being targeted for their increased cortical plasticity potential in gene therapy trials.^2^

AOSLO has started to be utilized in ACHM gene therapy clinical trial schedules and it will be some time before we are able to determine if this imaging modality is able to detect or predict therapeutic benefit and therefore aid patient selection. AOSLO has proven itself a non-redundant imaging modality with uncorrelated AOSLO and OCT metrics, and the detection of progressive cone loss when other imaging modalities, such as OCT and autofluorescence, are unable to detect changes.^16^^,^^20^^,^^39^ The gold standard for image analysis is currently manual counting of cones, where high interobserver reliability has been reported.^40^^–^^45^ There have been gradual improvements in automated techniques to reduce the technical, time, and cost burden of both image montaging,^46^^,^^47^ and cone counting in healthy retinas,^48^^–^^50^ and more importantly disease states, such as Stargardt disease and retinitis pigmentosa, which are more challenging to assess by automated systems.^51^

## Conclusions

This is the first longitudinal assessment of *CNGA3*-ACHM retinas showing stable foveal cone mosaics over time. The stability of PCD within patients over the follow-up period and the large variability within the cohort means an age cutoff recommendation for potential therapies is difficult to determine. The details of how AOSLO imaging will be used for trial end points or predictive factors in interventional research is yet to be fully elucidated as it has only recently started to be used in clinical trials.

## Supplementary Material

Supplement 1
