# Supplementary material for: Longitudinal Imaging of the Foveal Cone Mosaic in CNGA3-Associated Achromatopsia
Source: Invest Ophthalmol Vis Sci. 2024 Oct 4;65(12):6. doi: 10.1167/iovs.65.12.6 (PMC11460564; doi:10.1167/iovs.65.12.6)
Supplement: Supplement 1 [file iovs-65-12-6_s001.pdf]

Supplementary Table 1: Patient demographics at baseline and genotype

Subject ID	Sex	Age (years)	*CNGA3* (NM_001298.3) Variant 1		*CNGA3* (NM_001298.3) Variant 2	
			c.DNA change	Protein change	c.DNA change	Protein change
MM_0014*	Female	34.72	c.848G>A	p.Arg283Gln	c.667C>T	p.Arg223Trp
MM_0015*	Female	28.08	c.848G>A	p.Arg283Gln	c.667C>T	p.Arg223Trp
MM_0064	Female	23.84	c.1694C>T	p.Thr565Met	c.661C>T	p.Arg221Ter
MM_0170	Male	16.70	c.1642G>A	p.Gly548Arg	c.67C>T	p.Arg23Ter
MM_0385	Male	37.70	c.1228C>G	p.Arg410Trp	c.1228C>G	p.Arg410Trp
MM_0386	Female	22.21	c.1580T>G	p.Leu527Arg	c.1805G>A	p.Gly601Glu
MM_0398	Female	14.40	c.811C>T	p.Pro271Ser	c.829C>T	p.Arg277Cys
MM_0418	Male	18.32	c.661C>T	p.Arg221Ter	c.661C>T	p.Arg221Ter
MM_0446	Female	50.99	c.67C>T	p.Arg23Ter	c.67C>T	p.Arg23Ter
MM_0458	Female	19.27	c.725A>C	p.His242Pro	c.1773_1776del	p.Tyr591Ter

*Siblings. All patients previously reported[15] apart from MM_0458.
